# Application of integrated production and economic models to estimate the impact of Schmallenberg virus for various beef suckler production systems in France and the United Kingdom

**DOI:** 10.1186/s12917-014-0254-z

**Published:** 2014-10-26

**Authors:** Didier Raboisson, Agnès Waret-Szkuta, Jonathan Rushton, Barbara Häsler, Pablo Alarcon

**Affiliations:** Université de Toulouse, INP, ENVT, UMR 1225, IHAP, F-31076 Toulouse, France; INRA, UMR 1225, IHAP, F-31076 Toulouse, France; Veterinary Epidemiology Economics and Public Health Group, Royal Veterinary College, London, UK; Leverhulme Centre for Integrative Research on Agriculture and Health, Royal Veterinary College, London, UK

**Keywords:** Schmallenberg virus, Beef suckler, Production models, Gross margin, Partial budget, France, United Kingdom

## Abstract

**Background:**

Schmallenberg virus (SBV) was first detected in November 2011 in Germany and then rapidly spread throughout Europe. In beef suckler farms, clinical signs are mainly associated with reproductive disorders, particularly in late gestation, and intransient and non-specific symptoms, namely diarrhea, inappetence and fever. The objectives of this study were to develop models that simulate the production of different beef suckler systems in the United Kingdom (UK) and France and to use these models to estimate, through partial budget analyses, the farm-level economic cost of SBV under two disease impact scenarios, namely high and low impact. The probability for a farm to be in the high or low scenario depends, among other, on the high, low or nil vectorial activity for a given period and location and on the period(s) of sensitivity of the animals to the disease.

**Results:**

Under the high impact scenario, the estimated SBV impact ranged from 26€ to 43€ per cow per year in France and from 29€ to 36€ per cow per year in the UK. It was approximately half of this amount in the low impact scenario. These financial impacts represent 5 to 16% of the gross margin, depending on the country, impact scenario and livestock system considered. Most of the SBV impact originates from the costs of the steers and heifers not produced. Differences identified between the systems studied mainly stem from differences among the value of the steers or heifers sold: SBV impact is higher for British autumn calving systems compared to spring calving, and for French farms with calving and fattening activities compared to farms with only a single, annual calving activity.

**Conclusions:**

This study shows the usefulness of integrated production and economic models to accurately evaluate the costs of diseases and understand which factors have major impacts in the different systems. The models stand as a useful basis for animal health professionals when considering alternative disease control measures. They are also a farm accounting tool for estimating disease impact on differing production practices, which creates the necessary basis for cost-effectiveness analysis of intervention strategies, such as vaccination.

**Electronic supplementary material:**

The online version of this article (doi:10.1186/s12917-014-0254-z) contains supplementary material, which is available to authorized users.

## Background

Schmallenberg virus (SBV) was first detected in November 2011 in Germany [1]. It affects ruminant animals and appears mainly transmitted by insect vectors of the *Culicoides spp.* group and vertically *in utero* [2-4]. A transmission by bull semen was also recently observed [5]. Following expansive spread in various European countries, the virus was officially declared endemic in Belgium, France, Germany, Italy, Luxembourg, the Netherlands, Spain, Switzerland and the United Kingdom (UK) by the end of May 2012. In beef suckler farms, clinical signs are mainly associated with reproductive disorders. Depending on the time of infection, abortion, stillborn animals, premature deliveries and various intra-uterine congenital malformations may occur [6,7]. Schmallenberg virus has been detected in malformed foetuses, stillborn lambs or lambs born at term but with signs of neurological disorders, such as blindness, deafness, recumbency, an inability to suck and convulsions [7,8]. In adult cows, the acute infection can result in transient and non-specific symptoms, like diarrhea, inappetence, fever, and a reduction in milk yield, usually followed by a full recovery [1,9]. Such acute infections cause production losses in terms of animals and milk yield and require additional expenditures for palliative treatment of affected animals. Trade or movement regulations may be a further economic cost for farmers, because of immobilisation on infected animals and extra costs due to specific export requirements to SBV-free countries.

In order for beef producers to make an informed decision on a potential intervention investment to control a disease like SBV, it is essential to understand the trade-off between intervention costs and disease losses that can be avoided. This depends on the type of production system which in turn determines the characteristics of outputs and inputs and is associated with specific management decisions that rule reproduction and/or replacement decisions. Moreover it is linked to husbandry practices that influence the magnitude of losses and expenditures associated with disease. Thus, economic impact is determined with more accuracy when production systems are accounted for and when the production factors that cause the highest costs related to disease can be identified. Since France and the UK have herds of 3.9 and 1.5 million beef cows, respectively, and together account for 45% of the European beef cow herd, they are the focus of the present study.

The purpose of this work was to estimate the economic impact of SBV at farm-level for the most common beef suckler production systems of the UK and France. The objectives were 1) to develop beef suckler production models and define associated gross margins, 2) to calculate the partial budget for SBV in the UK and France, and 3) to investigate potential differences in model variables and disease estimates between the two countries.

## Methods

### Overview

For this research, the most typical beef suckler production systems in the UK and France were identified. They were modelled in Microsoft Excel to simulate the within-farm population dynamics and to estimate the annual gross margin (a measure of profitability) of each system. The annual gross margins obtained were compared with the respective published gross margins for validation purposes. Schmallenberg disease parameters were then included in the production models. A partial budget analysis was used to compare the extra costs and benefits of farm-level infections. Partial budget analyses included new costs, revenue foregone, costs saved and new revenue due to SBV. Values for the disease parameters were obtained from existing literature and by expert opinion consultation. Sensitivity analyses were conducted to assess the variability of the disease impact for different combinations of disease parameter values. Details on the method can be found elsewhere (Häsler B., Alarcon P., Raboisson D., Waret-Szkuta A., Rushton J., unpublished observations).

### Beef suckler production models

Available benchmarking data and expert opinion were used to identify the most common and representative beef suckler systems in the UK and France. In total, four production systems were identified for the UK and five for France (Additional file 1: Table S1).

For the UK, the systems were differentiated based on the geographic location (less favoured areas being upland *vs* lowland) and the calving season (spring *vs* autumn) and labelled taking into account these two factors (e.g. ‘lowland_spring’ for lowland systems with spring calving). In France, systems were based on the link between breed, area and husbandry practices. The Charolais, Limousin and Salers systems are located in Massif Central (centre of France) while the Blonde d’Aquitaine systems are in the South of France. All four systems represent farms specialised in calving activity (coded as Charolais_Calving, Limousin_Calving, Salers_Calving and Blonde_Calving), i.e. they sell six to 10-month-old weaned non-fattened calves for fattening (mostly to Italy). The fifth beef suckler model represents the Charolais calving and fattening farms (coded as Charolais_Fattening) in north-west France. In all systems, first calving mainly occurs at three years old, pasture (grass) is used in summer and cattle are housed in barns during winter. All the males and females are sold, except some females which are kept for replacement (i.e. they are raised on the farm until first calving). The Charolais_Calving and Limousin_Calving match the UK beef lowland spring calving model. The Salers_Calving matches the UK beef suckler upland spring calving model and the Charolais_Fattening the UK beef suckler lowland autumn calving model.

The production models simulated a one year production cycle by quantifying the different animal inputs and outputs (e.g. number of steers sold, number of heifers replaced, etc.). Benchmarking data from different independent sources based on farm surveys and actual expenditures made by farmers were used for both the UK [10-14] and France [14]. These publications were complemented by other sources such as the authors’ expertise and published statistics on market prices as required. For example, to disaggregate feed costs in France by the different class of animals the authors’ professional judgment was necessary as data were solely available for the whole farm. Production models included (i) revenue from sales of heifers and steers, (ii) replacement costs, (iii) feeding costs, (iv) veterinary and medicine costs and (v) other variable costs, such as bedding costs (Additional file 1: Table S2). Key differences between the French and British systems were as follows: heifers are commonly purchased in the UK whereas, in France, they are raised on-farm; disposal costs are paid by a tax at slaughter in France, but by farmers in the UK; the cost of forages used for calves in France is relevant, because some French farmers sell heavy 12–18 month old calves directly to slaughterhouse (an uncommon practice in the UK).

### Estimation of annual gross margins

The production models were used to estimate the annual gross margin for the different production systems (1):

1 $$ Gross\; margin= Revenue- Replacement\; costs\; and\; breeding\; depreciation- Feed\; costs- Veterinary\; costs- other\; variable\; costs $$

The revenue and costs calculated are listed in Table 1. Details of calculations are reported in Additional file 1: Table S2. All data used for the development of production models and gross margin analyses are listed in the Additional file 1: Tables S3 and S4. The economic data was also obtained through benchmarking, literature and authors judgment when no data was available.

**Table 1:** **Economic impact (in €) of Schmallenberg virus (SBV) for three types of beef suckler farms in France**

		**Charolais Calving**		**Salers Calving**		**Charolais Fattening**	
		**HI**	**LI**	**HI**	**LI**	**HI**	**LI**
Additional expenditure	Veterinary assistance on cows that have dystocia due to SBV	73	37	75	38	73	37
	Treatment of cows that need caesarean due to SBV dystocia	7	3	7	3	7	3
	Treatment of cows that have clinical SBV episodes	26	0	26	0	26	0
	Treatment of cows that have aborted due to SBV	60	30	60	30	60	30
	SBV testing of aborted foetuses, stillborn or malformed calves	1	0	1	0	1	0
	Cost of purchasing and raising heifers for replacement	75	38	126	63	76	38
Revenue forgone	Steers not sold	1,810	910	1,548	778	2,533	1,273
	Heifers not sold	1,866	937	1,657	832	2,693	1,353
	Cows that die	44	22	46	23	44	22
Sum of costs		3,961	1,977	3,546	1,769	5,512	2,757
Expenditure saved	Concentrate feed saved on steers and heifers not produced	256	137	453	228	512	261
	Concentrate feed saved on cows that die or are culled	54	27	43	22	54	27
	Bedding costs saved	162	81	63	32	194	97
	Miscellaneous costs saved	117	59	72	36	153	77
	Cow vaccines saved	1	1	1	1	1	1
	Calf vaccines saved	58	29	59	29	58	29
	Cow worming saved	1	1	1	1	4	2
	Calf worming saved	5	3	5	5	5	3
Extra revenue	Revenue from cows culled due to SBV abortion	230	115	160	80	200	100
Sum of benefits		884	451	857	442	1,181	595
NET TOTAL SBV COST (€)/HERD		3,077	1,526	2,689	1,347	4,256	2,162
NET TOTAL SBV COST (€)/COW		30.7	15.3	26.9	13.5	42.5	21.6
Range of plausible values (€/cow)		8-99	0-14	7-84	0-15	11-135	0-22

Ranges of plausible values are defined with minimum and maximal parameters, as listed in Table 2.

*HI*, high impact disease scenario; *LI*, low impact disease scenario.

### Assessment of SBV disease impact using partial budget models

First, on the basis of a literature review, the biological effects of SBV in beef suckler cattle were identified (Table 2). Further, common management practices were discussed and assumptions made regarding farmers’ reactions to disease without considering labour (Additional file 1: Table S5). For instance, it was assumed that SBV will result in extra culling because farmers will not use animals with reproductive disorders for breeding again. Although there are anecdotal reports that SBV may cause infertility in cows, there is no robust scientific evidence available yet about such effects so infertility problems were excluded from this study. The diversity of factors involved in infertility proposes a challenge for farmers and experts to establish a causal effect of SBV infection. Second, the disease parameters were introduced in the production models. The differences obtained between gross margin parameters of disease and no disease situations were calculated. For example, the proportion of abortions due to SBV changed the number of calves born, which then resulted in lower revenue from calves sold. For new cost items, new parameters were created in the model, such as “cost of caesarean” (number of caesarean * costs of one caesarean) or “cost of SBV testing” (number of foetuses tested * cost of one SBV test).

**Table 2:** **Parameters and values used for a high impact and low impact Schmallenberg virus disease scenario**

**Parameters**	**Scenario 1 High impact**	**Scenario 2 Low impact**	**References**	**Reasoning**
Number of calves stillborn or malformed due to SBV out of 100 calves born	1-10 most likely = 2	0-1 most likely = 1	[15] and expert opinion	Martinelle *et al.* 2012 [15]: median SBV morbidity rate in calves was 2% ; the minimum reported by Martinelle *et al.* [15] was taken as the lower range value and the median value plus one standard deviation as the upper range value.
Number of cows with dystocia out of 100 cows giving birth to a stillborn or malformed calf due to SBV	30		[16,17] and expert opinion	Baseline dystocia rates in UK are 6.9% in heifers and 2% in cows with abnormal presentations being the cause in 19.8% on average. With an increased proportion of malformations, dystocia rate was assumed to be higher.
Number of cows that need caesarean out of 100 cows with dystocia due to SBV	5-7 most likely = 6		[18,19] and expert opinion	The proportion of caesareans conducted in the case of dystocia was reported to be between 5 and 7%.
Number of cows with clinical episodes due to SBV out of 100 cows in a herd	3-31 most likely = 7.5	0	[15] and expert opinion	Martinelle *et al.* 2012 [15]: Median SBV morbidity rate in cattle was 7.5%. The minimum reported by Martinelle *et al.* [15] was taken as the lower range value and the median value plus one standard deviation as the upper range value.
Number of cows that require treatment out of 100 cows with clinical episodes due to SBV	10		Expert opinion	This figure reflects the regular need for treatment of beef sucklers in the UK presented with unspecific diarrhoea, fever, general depression and/or inappetence.
Number of cows with SBV abortions out of 100 cows in a herd	0-2 most likely = 2	0-1 most likely = 1	Expert opinion	The proportion of abortions due to SBV is uncertain (lack of studies). Experts agreed on these approximated figures based on abortion rates seen in other diseases.
Probability of an aborted foetuses, stillborn, malformed and calves culled to be tested for SBV	0.05		Expert opinion	Investigation of abortions is recommended if incidence >3% in a herd per year or if several abortions occur in quick succession (http://www.defra.gov.uk/ahvla-en/files/pub-cattle-abortion.pdf). Due to the absence of “abortion storms” due to SBV and farmers suspecting the disease, it is assumed that only a small proportion submit aborted foetuses, stillborn or malformed calves to be tested for SBV.
Number of cows that die due to calving difficulties out of 100 cows with dystocia	10		[17,18] and expert opinion	Day and Meijering report mortality rate due to dystocia as 3.5% on average, and 16.7% for a clinical case observation. Given that SBV causes malformations, the mortality rate is assumed to be on higher than the reported average.
Number of aborted cows that will need to be replaced out of 100 cows with abortions	10		Expert opinion	It was assumed that only in a small proportion of cows the reproductive system will be affected such that the cow is not able to breed anymore and will therefore be replaced.

Finally, the differences of the gross margin were compared using a partial budget analyses (2):

2 $$ Net\;SBV\; economic\; cos{t}_i=\left( Costs\; save{d}_i+ New\; revenu{e}_i\right)-\left( New\; cos t{s}_i+ Revenue\; forgon{e}_i\right) $$

*Net SBV economic cost r*epresents the economic impact of the disease and *i* a defined disease scenario.

For data on the within-herd SBV incidence, the incidence of various disease effects (e.g. rate of abortion, percentage of cows with clinical signs) and the magnitude of those effects or consequences (e.g. proportion of cows with dystocia that will need caesarean) are sparse but sufficient to consider two impact scenarios:

- Scenario 1: High impact in a herd that is highly susceptible to disease, which may be for example a management system where the susceptible gestation period falls into a season of high vector activity.
- Scenario 2: Low impact in a herd that is less susceptible to disease, which may be for example a management system in an area with low vector density or where the gestation period falls into a season with low vector activity.

For each scenario, input parameters were defined as summarised in Table 2 to calculate the partial budget. In addition to the values derived from the scientific literature, the input values for the model were discussed and agreed on during an expert workshop as described below. For the most variable and uncertain parameters, minimum, most likely and maximum values were agreed upon. In brief, the three parameters that differed between the high and low impact scenario were (i) the percentage of stillborn and malformed calves, (ii) the percentage of cows with clinical episodes due to SBV and (iii) the percentage of cows with abortion (Table 2).

### Software, input values, sensitivity analysis, and validation

All models were developed and run in Microsoft Excel 2010 (Microsoft Corporation). Apart from the parameter values derived from published literature, a workshop with 10 experts representing members of the Schmallenberg surveillance team at the Animal Health Veterinary Laboratories Agency, industry representatives, veterinary clinicians and academic researchers was held to present and discuss the structure of the production models, input variables and assumptions. Before the meeting experts were requested to give their opinion on the values of some of the disease parameter for high and low impact scenarios. The different values obtained were then presented to the experts during the workshop. For those parameters with major differences a discussion was stimulated to agree on the value. Annual gross margins obtained were compared with the respective published gross margins for validation purposes. The sensitivity of the model to a simultaneous change of the variable percentage of stillborn and malformed calves due to SBV and the variable percentage of cows with late abortions due to SBV was tested by changing their values from 0 to 5% and from 0 to 3.5% respectively, as these two parameters were defined as the most important disease factors by the workshop participants. The models were also run with all lowest and all highest values to estimate the range of disease impact.

For purpose of comparison and clarity, all economic results are presented in euros (1€ = £0.8128, as consulted on the 20^th^ of May 2014).

## Results

### Production models and gross margin

**Figure 1:**
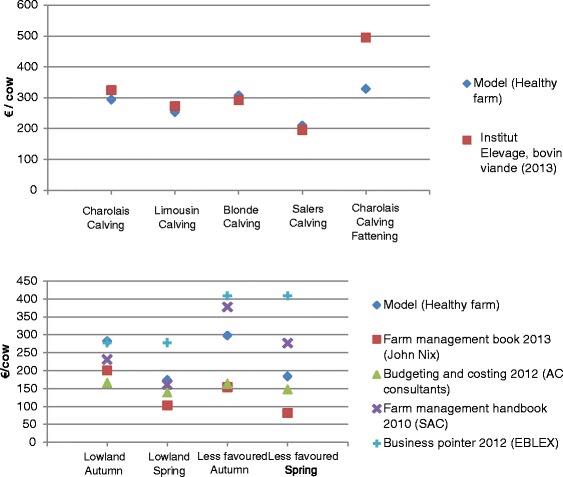
**Gross margin results for SBV free beef suckler farms in France (up) and in the UK (down) and comparison with other gross margin analyses existent in the literature.** Institut Elevage, bovin viande (2013) = [14]; Farm management book 2013 = [11]; Budgeting and Costing 2012 = [12]; Farm management handbook 2010 = [10]; Business pointer 2012 = [13].

**Figure 2:**
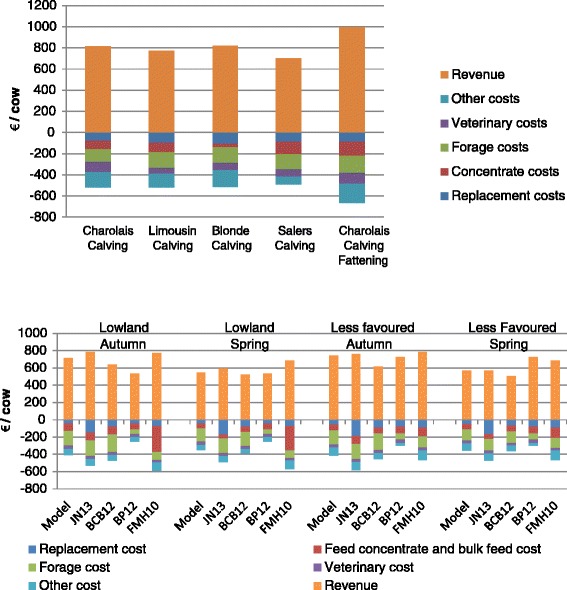
**Break down of the gross margin for SBV free 5 types of beef suckler production systems in France (up) and for 4 types of beef suckler production systems in the UK (down).** JN13 = John Nix 2013 = [11]; BCB12 = Budgeting and Costing Book 2012 [12]; BP12 = Business pointer 2012 = [13]; FMH2010 = Farm management handbook 2010 = [10].

Summary results of the gross margin analyses are presented in Figures 1 and 2. The detailed structure and results of the Charolais_Calving production models and gross margin analyses of non SBV-infected farms are presented in Additional file 2: Tables S1 and S2.

In France, the model gross margins obtained for Charolais_Calving, Limousin_Calving, Blonde_Calving, Salers_Calving and Charolais_Fattening were 293€, 253€, 307€, 209€ and 329€ per cow per year, respectively (Figure 1). The lower gross margin observed for Salers_Calving is due to the reduced revenue, and the higher gross margin observed for Charolais_Fattening is due to higher revenue in spite of higher feeding costs (Figure 2). All results match the reference ones, except a 33% lower gross margin in the present study compared to reference for Charolais_Calving. The sum of production costs is in the same range, and the difference mainly originates from the feeding cost.

For the UK, the model gross margin obtained for Lowland_autumn, Lowland_spring, LessFavoured_Autumn and LessFavoured_Spring were 281€, 173€, 297€ and 184€ per cow per year, respectively (Figure 1). The main differences observed in Lowland_autumn and LessFavoured_Autumn between the model gross margin and the industry gross margin as calculated by the industry (Business pointer 2012), are due to the estimation of revenue from selling calves (Figure 2). The difference in revenue is mainly caused by the way calf weight is estimated. The main differences observed in Lowland_Spring and LessFavoured_Spring between the model gross margin and the industry gross margin are explained by the forage cost estimation.

### Impact of SBV

The net SBV economic cost of SBV (in €/cow/year) for an average French beef suckler farm was estimated at 30.7€ and 15.3€ for Charolais_Calving farms, 30.1€ and 14.5€ for Limousin_Calving farms, 31.9€ and 15.4€ for Blonde_Calving farms, 26.9€ and 13.5€ for Salers_Calving farms, and 42.5€ and 21.6€ for Charolais_Fattening, for the high and low impact scenario, respectively (Table 1). Results of Limousin_Calving and Blonde_Calving are very close to that of Charolais_Calving, so only those for Charolais_Calving are reported here in detail. The costs mainly accrued from steers and heifers not sold (at least 90% of the sum of costs), whatever the system and the scenario (high or low impact).

For the UK, the net SBV economic cost (in €/cow/year) for an average farm was estimated at 34.8€ and 17.5€ for Lowland_Autumn farms, 29.3€ and 14.7€ for Lowland_Spring farms, 36.4€ and 18.3€ for LessFavoured_Autumn farms and 30.0€ and 15.0€ for LessFavoured_Spring farms, for the high and low impact scenario, respectively (Table 3). For France, the new costs and revenue foregone accrued mainly from the revenue foregone from steers and heifers not sold, regardless of the system and scenario (over two thirds of the sum of costs).

**Table 3:** **Economic impact of Schmallenberg virus (SBV) for the 4 types of beef suckler farms in the UK**

		**Lowland_Autumn**		**Lowland_Spring**		**Less Favoured_Autumn**		**Less Favoured_Spring**	
		**HI**	**LI**	**HI**	**LI**	**HI**	**LI**	**HI**	**LI**
Additional expenditure	Veterinary assistance on cows that have dystocia due to SBV	60	32	64	32	64	32	64	32
	Treatment of cows that need caesarean due to SBV dystocia	7	4	7	4	7	4	7	4
	Treatment of cows that have clinical SBV episodes	10	0	10	0	10	0	10	0
	Treatment of cows that have aborted due to SBV	255	127	255	127	255	127	255	127
	SBV testing of aborted foetuses, stillborn or malformed calves	1	1	1	1	1	1	1	1
	Cost of purchasing and raising heifers for replacement	421	212	421	145	449	140	449	140
	Disposal costs of dead calves and foetus due to SBV	285	145	289	212	279	225	279	225
Revenue forgone	Steers not sold	1,578	771	1,168	587	1,583	796	1,209	608
	Heifers not sold	1,393	700	1,085	545	1,452	730	1,122	563
	Cows that die	33	18	36	18	36	18	36	18
Sum of costs		4,001	2,009	3,334	1,670	4,135	2,319	3,431	1,719
Expenditure saved	Concentrate feed saved on steers and heifers not produced	109	55	54	27	117	59	74	37
	Concentrate feed saved on cows that die or are culled	84	42	54	27	64	32	54	27
	Bulk feed saved	49	25	44	22	20	10	39	20
	Forage saved on cows culled	0	0	0	0	0	0	0	0
	Bedding costs saved	89	44	78	39	122	62	108	54
	Miscellaneous costs saved	47	23	39	20	47	23	39	20
	Cow vaccines saved	0	0	0	0	0	0	0	0
	Calf vaccines saved	5	2	5	2	5	2	5	2
	Cow worming saved	0	0	0	0	0	0	0	0
	Calf worming saved	0	0	0	0	0	0	0	0
Extra revenue	Revenue from cows culled due to SBV abortion	132	64	129	64	116	58	116	58
Sum of benefits		514	256	405	201	491	242	436	215
NET TOTAL SBV COST (€)/HERD		3,487	1,753	2,929	1,470	3,644	1,829	2,996	1,503
NET TOTAL SBV COST (€)/COW		34.9	17.5	29.3	14.7	36.4	18.3	30.0	15.0
Range of plausible values (€/cow)		9-106	0-17	7-89	0-15	10-109	0-18	7-90	0-15

Ranges of plausible values are defined with minimum and maximal parameters, as listed in Table 2.

*HI*, high impact disease scenario; *LI*, low impact disease scenario.

Sensitivity analyses were performed for two of the most sensitive and uncertain disease parameters. The variations of the net SBV economic cost obtained during these sensitive analyses are illustrated in Additional file 2: Table S3. The range from the best case (using minimum values for all disease inputs as defined in Table 2) to the worst case (using maximum values for all disease inputs as defined in Table 2) for the low and high impact scenario are reported in Tables 1 and 3 (in the row of ‘range of plausible values’). Results of the sensitivity analyses and the range found from best to worse case of Limousin_Calving and Blonde_Calving are very close to those of Charolais_Calving for which details are provided here. Similarly, sensitivity analyses and ranges from best to worse case of Lowland_Autumn and LessFavoured_Autumn were close, as were results for Lowland_Spring and LessFavoured_Spring.

### Comparison of gross margins with and without SBV

**Figure 3:**
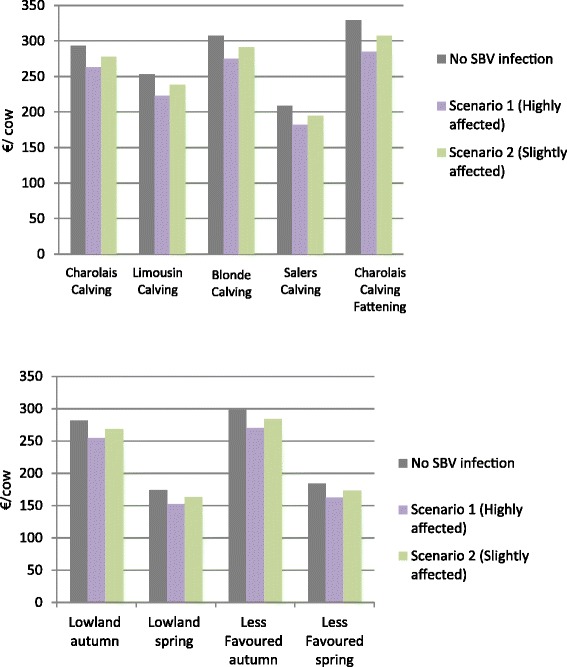
**Gross margins (€/cow) for not SBV affected, highly and slightly SBV affected beef suckler farms in France (up) and in the UK (down).**

The impact of SBV on the farm gross margins is shown in Figure 3. The figures illustrate the gross margin expressed as € per cow per year, respectively, for a farm not infected with SBV, highly affected and slightly affected. The reductions in gross margins for the high impact scenario are 10% in Charolais_Calving farms (FR) and Blonde_Calving farms (FR), 12% in Limousin_Calving farms (FR), in Lowland_Autumn farms (UK) and in LessFavoured_Autumn farms (UK), 13% in Salers_Calving (FR) and Charolais_Fattening (FR) farms and 16% in Lowland_Spring farms (UK) and LessFavoured_Spring (UK) farms. Percentages are reduced by two fold in the low impact scenarios.

## Discussion

The present study used partial budget and gross margin analyses in combination with production models to estimate the economic impact of SBV in different beef suckler livestock system. The main advantage of combining production models and partial budget analysis is that it exposes the cascade effect that the disease may have on the production and the farm performances (e.g. extra dystocia caused because of stillborn or malformed calves due to SBV). Although the time frame chosen for this study was one year, the modelling approach complements the dynamic population of the herd and allows a precise quantification of performance changes that would not be possible through a partial budget analysis alone [20,21]. Moreover, the calculation of the gross margin in the production model allows a direct validation of the model with benchmarking data and therefore provides a solid foundation for disease impact studies. The use of gross margin analysis also proved useful to understand the impact of the disease on the profitability of each system. However, the models do not take into account the medium or long term consequences of the SBV infection. Such predictions could be made by inclusion of behaviour assumptions in the models (e.g. on variation in management over time), predictions on price developments and, most importantly, the epidemiology of the disease and related effects. While a farm’s replacement policy may change in the long term, the beef industry is mainly focused on the production of one calf per year per cow and it is therefore intuitive to estimate the disease consequences for a one year production cycle. Most of the carry-over effects to the next following year(s) were integrated within the studied one-year cycle period, in particular for extra culling and extra replacement. Similarly mortalities or abortions during the studied year for animals that would have been sold in the following year in case of no disease were accounted for in the studied year.

For France, the disease impact is similar for the three main systems (Charolais_Calving, Limousin_Calving and Blonde_Calving), slightly lower for Salers_Calving and slightly higher for Charolais_Fattening - independent of the high or low impact scenario considered. For UK, the results show a slightly increased impact of SBV for autumn calving compared to spring calving in UK, both for the low and high impact scenarios. This is mainly attributable to the higher revenue usually obtained on autumn calving from the calves sold. Thus, for both countries, it was found that the higher the revenues in the gross margin, the higher the SBV impact. The revenue greatly depends on the selling prices of the heifers and steers (which depend on breed, age of selling and season of selling). The differences in SBV impact between the different livestock systems mainly come from the differences in revenues between the systems. The fact that French Charolais_Fattening has the higher SBV impact may originate from the period of calving in autumn, as suggested by the higher impact in UK for autumn calving systems as compared to spring calving systems. Yet, this is probably more linked to the fattening activity than to the calving period. The net revenue per cow is higher when calves are fattened compared to when weaned calves are sold, and the loss of a calf due to SBV has consequently a higher impact. Indeed, in all the systems considered, the major SBV cost for a beef suckler farm is associated with the losses due to steers and heifers that could not be sold because of the disease. Yet, the SBV impact estimation may have been overestimated for Charolais_Calving since the present results account for idle production capacity but farmers could replace the lost weaned calves entering the fattening unit with purchased ones. Other major costs in beef suckler herds are those accrued from the cost of purchasing replacement heifers (in UK and to a lesser extent, in France) and the disposal cost of dead or culled animals (in UK only).

The difference between calculated and reference gross margins observed for the French Charolais_Calving system is due to the feeding costs. This difference likely originates from the variability among farms within (i) the age at weaning, (ii) the cost of feeding cows and calves in barns (higher part of the year compared to calving systems), and (iii) the distribution of the feeding costs between forage and concentrates (various indoor diets, with more or less forage and concentrates). The farming systems yet remain ranked according to the gross margin in the same way as in the references used (lower gross margin for extensive system, higher gross margin for fattening system). For the autumn calving herds in the UK, the revenue from calves sold was slightly higher than the industry estimates. In the present results and in accordance with existing literature [11], it is considered that autumn calving systems produced calves that are sold at much higher weight (average 358 kg) than spring calving systems (average 275 kg). The industry benchmarking [13], do not differentiate autumn and spring calving systems (average calf weight is 279 kg). In addition, calf price used in the present model is in accordance with [11] and is slightly higher than the one used by the industry benchmarking [13]. Furthermore, industry gross margins are higher due to lower forage cost, which were not used to calculate impact of disease in this study. Therefore, the production model developed is believed to reflect the industry gross margin for the different beef suckler systems.

One of the main limitations of this study was the lack of data available in the literature on SBV disease effects, which may be partly due to a lack of reporting and the absence of incentives for reporting. Most of the published scientific literature described the situation on SBV affected farms, but only in some exceptional cases compared them to non-affected farms or previous years before SBV emergence. As a result, attribution of disease estimates was not possible from those studies so experimental or epidemiological studies comparing affected and non-affected farms are needed to obtain more accurate disease estimates. The disease estimates used in this study were derived from scientific publications where ever possible and complemented by expert opinion consultation. Sensitivity analyses on disease estimates were used to account for this uncertainty and demonstrate the influence of the most uncertain input values used.

The present work estimates the net SBV economic costs under French and British conditions, for nine production systems and under two scenarios. The disease impact may reach up to 5 to 17% of the gross margin in the worst case, depending on the system, the country and the impact scenario. SBV may consequently slightly change the economic performance of some farms. The disease impact differs more between livestock systems within a country than between countries.

These results are of great interest for farmers and veterinarians in field. They also may be useful for decision makers as part of a decision making process. When using the results, three considerations apply. First, the present estimations represent the total cost of the SBV at farm-level and not the avoidable costs. Thus, if seeking a trade-off with the cost of vaccination, the current results may be used but acknowledging the gap between total costs and avoidable costs. The best way to evaluate such a trade-off would be to perform an economic efficiency analysis of possible SBV vaccination strategies, with the efficacy and price of the vaccines known. Yet, because of the differences in institutional factors between the two countries, such as veterinarian services or mean herd size, the control of SBV may depend on different production strategies in France and the UK, even if no noticeable difference in SBV impact is observed between France and the UK in the present work. Second, the present estimations are made for a one year cycle, and may misrepresent the medium or long term consequences of the SBV. Third, the use of the present results to make a first, raw calculation of the national impact of SBV is possible by multiplying the SBV impact (nil, low or high) by the number of farms or cows concerned. Yet the set of possible situations depends on (i) the high, low or nil vectorial activity for a given period and location and (ii) on the period(s) of sensitivity of the animals to the disease. For instance, knowledge of the production system suggests that autumn and early winter calving herds (i.e. UK autumn calving systems and French Calving_Fattening) should be considered in the high impact scenario. On the contrary, the spring calving systems are more likely to follow the results of the low impact scenario, although the impact could be nil if the period of mid-gestation is distinct from that of vector activity (winter). Moreover, the impact is more likely to be high for an infection of a SBV naïve herd although it may remain low and perhaps nil in case of re-infection in endemic situation. Information regarding SBV immunity strength and duration is needed to estimate the probability of high or low scenario under endemic situation. Whatever the case, because of the numerous situations regarding the vectorial activity and cow infection characteristics, calculating the national impact of SBV based on the current work is possible (albeit challenging) and remains open to further research.

## Conclusions

For the high impact scenario, the net SBV economic cost was estimated from 26€ to 43€ per cow per year in France and from 28€ to 37€ per cow per year in the UK (5% to 16% of the gross margin). It was half in the case of the low impact scenario. High and low impact scenarios might depend on the gestation period at which infection occurs, the vector density in each system, the immunity of the herd and other factors, such as breed. Therefore farms with calving periods around autumn might be more likely to be highly affected. Most of the SBV impact originates from the costs related to the sub-optimal performance of herds. Differences observed between the systems studied mainly arise from the differences among the value of the steers or heifers sold. Even though total SBV costs, but not unavoidable costs are estimated here, the present work provides a useful basis to evaluate the economic efficiency of SBV control measures at farm-level.

### Availability of supporting data

The data sets supporting the results of this article are included within the article and its additional files.

## Additional files

Additional file 1:
**Production types and details parameters and calculations.**
Additional file 2:
**Details results and sensitivity analysis.**
