# Supplementary material for: Application of integrated production and economic models to estimate the impact of Schmallenberg virus for various beef suckler production systems in France and the United Kingdom
Source: BMC Vet Res. 2014 Oct 26;10:254. doi: 10.1186/s12917-014-0254-z (PMC4221685; doi:10.1186/s12917-014-0254-z)
Supplement: Additional file 1: — Production types and details parameters and calculations. [file 12917_2014_254_MOESM1_ESM.doc]

Additional file 1

**Table S1: Description of the beef suckler production types in the United Kingdom (UK) and France (FR) considered in this study.**

|  | **Farm types** | **Description** |
| --- | --- | --- |
| FR | **Charolais_Calving** (Lowland Massif Central Charolais Calving activity) | Calves are mainly born between January and March. Sometimes heifers calve earlier. All calvings take place in barns. Calves are weaned and sold simultaneously in September/October at 8-10 month old. First calving occurs at 3 years old. The diets are based on hay and concentrates sometimes corn silage in winter and grass, sometimes hay in summer. Calves sometimes eat hay and concentrates in winter and always grass and concentrates in summer. Most of the concentrates are produced at the farm. |
| FR | **Limousin_Calving** (Lowland Massif Central Limousin Calving activity) | The same as Charolais_calving, except earlier weaning and selling (7-10 month old), slight differences in feed quantity and calves selling prices. |
| FR | **Salers_Calving** (Upland Massif Central Salers Calving activity) | Long period in barns (snow and cold). Calves are born between February and April and weaned / sold in September/November at 7-10 month old. All male calves are sold; females are sold if Charolais crossed is used. Overall age of cows is higher and replacement rate is lower than previous systems. Winter feed is based on hay, and few quantities of concentrates. High quality and quantity of grass in summer. Possibility to lose weight in winter and to re-gain it in summer. In summer, moderate to high quantity of concentrates are given to calves. All concentrates are bought. |
| FR | **Blonde_Calving** (Lowland Massif Central Blonde d’Aquitaine Calving activity) | All year around calving with high calving interval in most of cases. Calves are sold between 6 and 8 months old. Diets based on hay, corn silage and concentrates for winter. Grass used in summer, with other feed (hay) needed because of hot weather. High quality feed needed for this breed. No weight loss permitted. |
| FR | **Charolais_Fattening** (Lowland West-France Charolais Calving and Fattening activity) | Some calvings occur in August or in autumn. Possibility of a second calving peak in winter. Calves weaned early in winter and fattened on the farm indoor. Sold at 13-16 months old for slaughterhouse. Cost of feeding tends to be higher, because cows and non weaned calves eat more in winter during autumn calving compared to winter calving. Use of corn silage possible. Production of concentrates in farm for most of them. |
| UK | **Lowland_Autumn** (Lowland autumn calving farms) | Calves are born between August and October. Calves are sold in September/October the following year at 10-12 month old. Cows and calves are kept in covered yards with straw bedding during winter period. Therefore, extra amount of concentrates in the feed is required during that period. |
| UK | **Lowland_Spring** (Lowland spring calving farms) | Calves are born between February and May and are sold in autumn at 7-8 months old. Calves are mainly feed on grass in rich grazing lands. Cows are kept in straw yards over winter. |
| UK | **LessFavoured_ Autumn** (Less favoured areas autumn calving farms) | Less favoured areas are hills and uplands. Calves are born between September and November and sold at 10-11 month old. Cattle are raised in poor grazing areas and overwinter. A lower stocking rate and an extra amount of concentrates required. |
| UK | **LessFavoured_ Spring** (Less favoured areas spring calving farms) | Calves are born between February and May. They are then sold at 7-8 month old. Cattle are raised in poor grazing areas and therefore require a lower stocking rate. |

Table S2: Revenue and costs calculated in production models for beef suckler systems in France (FR) and the United Kingdom (UK). Input values are specific to each production system. Number and quantities of animals/products indicated in the equations are obtained from the production models.

| **Revenue and costs** | **Equations** |
| --- | --- |
| ***Revenue*** |  |
| FR-UK: Sales from steers | Total number of steers produced and sold * Live weight of a steer (kg)* Price (€) of calves per kg live weight |
| FR-UK: Sales from heifers | Total number of heifers produced and sold * Live weight of a heifer (kg)* Price (€) of calves per kg live weight |
| FR-UK: Sales from culled cows | Total number of cows culled * Cull price (€) per cow |
| ***Replacement cost*** |  |
| UK: Cost of purchasing replacement heifers | Total number of heifers bought for replacement * Net value of a replacement heifer (€/head) |
| FR : Costs of raising replacement heifers (after weaning) | Total number of heifers for replacement (not sold) * [Yearly cost of ration / heifer (€) year 1 (> age of weaning) + Yearly cost of ration / heifer (€) year 2 + Yearly cost of ration / heifer (€) year 3] |
| UK: Cost of disposal of dead heifers | Number of heifers that died * Disposal cost of an Heifer (€) |
| FR-UK : Cost of bull replacement | [((Price per bull (€) - Cull value of a bull (€)) / Life of a bull in years) /Cow:bull ratio]*Herd size |
| ***Feeding cost*** |  |
| FR-UK: Costs of concentrates used in cows | Total number of cows that calved *Concentrate (kg) per cow*price (€) per kg concentrate |
| UK : Costs of concentrates used in calves | (Total number of steers produced and sold + Total number of heifers produced and sold) * Concentrate per calf (kg) * Price of the concentrate use for calves (€/kg) |
| FR: Costs of food (concentrates and forages) used in sold steers and heifers | (Total number of steers produced and sold*(Concentrate (kg) per steer * Price per kg of concentrate use for steer/heifers (€) + Forage cost per steer/heifer per year indoor up to weaning + Forage cost per steer/heifer per year during fattening ) ) + (Total number of heifers sold *(Concentrate (kg) per steer * Price (€) per kg of concentrate use for steer/heifers + Forage cost per steer/heifer per year indoor up to weaning + Forage cost per steer/heifer per year during fattening )) |
| FR: Costs of concentrates used in heifers calves up to weaning | Total number of heifers needed for replacement * Concentrate per heifer sold (kg) * Price (€) per kg of concentrate use for calves |
| UK: Costs of bulk feed per cow per year | Total number of cows that calved * Cost of bulk feed/cow (€) |
| FR-UK: Cost of forage per cow per year | Total number of cows that calved * Forage cost/cow/year (€) |
| ***Veterinary and medicine costs*** |  |
| FR-UK: Veterinary costs | Total number of cows * Veterinary costs / cow (€) |
| UK: Costs of disposing dead cows | Total number of cows that died * Cost of disposing a dead cow (€) |
| UK: Costs of disposing dead calves | Total calves that died non due to SBV * Cost of disposing a dead calf (€) |
| ***Other variable costs*** |  |
| FR-UK: Bedding costs | Total number of cows * bedding used (ton/cow) * Price of bedding (€/ton) |
| FR-UK: Miscellaneous costs | Total number of cows * Miscellaneous cost /cow (€) |

Table S3: Parameters used to simulate different beef production systems in France

| Parameters | Symbol | Charolais_Calving | | Limousin_Calving | | Blonde_Calving | | Salers_Calving | | Charolais_Fattening | | References |
| --- | --- | --- | --- | --- | --- | --- | --- | --- | --- | --- | --- | --- |
|  |  | Value | Variability | Value | Variability | Value | Variability | Value | Variability | Value | Variability |  |
| ***Physical performance data*** |  |  |  |  |  |  |  |  |  |  |  |  |
| Proportion of barren cows | PrpBC | 0.065 | - | 0.059 | - | 0.066 | - | 0.027 | - | 0.061 | - | Calculated (1- (NPbs +MorC01+ MorC18)/100) |
| Calf mortality (0-1 months - %) | MorC01 | 6.0 | - | 4.6 | - | 6.3 | - | 4.0 | - | 6.0 | - | [1] |
| Calf mortality (1-8 months - %) | MorC18 | 1.9 | - | 1.5 | - | 2.0 | - | 1.3 | - | 1.9 | - | [1] |
| Numeric productivity (%) | NPbs | 86 | - | 88 | 88-90 | 85 | 83-85 | 92 | 94 | 86 | 88 | [2] |
| Replacement rate (%) | RRBs | 25 | 24-27 | 22 | 21-23 | 23 |  | 17 |  | 28 | 26-28 | [2] |
| Additional mortality of replacement heifers (%) | MH | 0.06 | - | 0.06 | - | 0.06 | - | 0.06 | - | 0.06 | - | UK data as proxy [3] |
| Male-female calf ratio | MFR | 0.5 | - | 0.5 | - | 0.5 | - | 0.5 | - | 0.5 | - | Assumption |
| Average live of a bull (years) | LiveB | 4 | - | 4 | - | 4 | - | 5 | - | 4 | - | [4] |
| Cow mortality (%) | MrtC | 1.5 | - | 1.5 | - | 1.5 | - | 1.0 | - | 1.5 | - | [1] |
| Cow bull ratio | CBR | 35 | - | 35 | - | 35 | - | 35 | - | 35 | - | [4] |
| Liveweight of a steer at selling point (kg) | LwSteer | 393 | - | 350 | - | 300 | - | 362 | - | 440 | - | [2] |
| Liveweight of a heifer calf at selling point (kg) | LwH | 355 | - | 325 | - | 270 | - | 340 | - | 410 | - | [2] |
| ***Animal economic data*** |  |  |  |  |  |  |  |  |  |  |  |  |
| Price per liveweight of a calf (€) | PrCal | 2.4 | 2-2.4 | 2.56 | 2,56-2,65 | 3.1 | 2,61-3,10 | 2.19 | 1,84-2,19 | 3 |  | [2] |
| Cow cull value (€) | CCull | 1,150 | - | 1,000 | - | 1,250 | - | 800 | - | 1,000 | - | Estimate from [5] |
| Price of a replacement heifer (€) | PrH | 1,500 | - | 1,500 | - | 1,500 | - | 1,500 | - | 1,500 | - | [5] |
| Price of a bull for replacement (€) | PrB | 1,500 | - | 1,500 | - | 1,500 | - | 1,500 | - | 1,500 | - | Assumption |
| Cull value of a bull (€) | BCull | 800 | - | 800 | - | 800 | - | 800 | - | 800 | - | Assumption |
| ***Feed parameters*** |  |  |  |  |  |  |  |  |  |  |  |  |
| Concentrate per cow (Kg) | ConCow | 150 | 100-200 | 125 | 90-150 | 150 | 100-200 | 75 | 50-100 | 210 | 150-250 | Assumption: 1 kg/d for 4-5, 3-4, 3-4, 2.5 and 4-5 months for systems from left to right, respectively |
| Concentrate per steer sold(Kg) | ConSteer | 400 | - | 290 | 200-370 | 141 | 125-170 | 400 | 350-450 | 460 | 433-485 | [2] and estimate selling weights |
| Concentrate per heifer calf sold (Kg) | ConHeifC | 361.3 | - | 269.3 | - | 126.9 | - | 375.3 | - | 428.6 | - | Estimated from ConSteer relative to weight at selling. |
| Price of cow concentrate (€/kg) | PrConCow | 0.175 | - | 0.25 | - | 0.125 | - | 0.28 | - | 0.125 | - | Based on half wheat half soybean meal and prices from [5] |
| Price of calf concentrate (€/kg) | PrConCalf | 0.175 | - | 0.25 | - | 0.125 | - | 0.28 | - | 0.125 | - |  |
| Forage cost/cow/year | CostForHa | 128.7 | - | 155.3 |  | 157 | - | 144.9 | - | 169.5 | - | Indoor, outdoor and outdoor with hay are 4/7/1, 4/8/1, 4/7/2, 4/7/2, 5/7/0, 5/6/1 months for systems from left to right, respectively |
| Diet price / heifer (€) in year 1 after weaning | DRH1 | 66.19 | - | 81.47 | - | 71.28 | - | 97.76 | - | 22.13 | - | Based on average DM intake and same food costs as steers ad cows |
| Diet price / heifer (€) in year 2 | DRH2 | 122.9 | - | 151.2 | - | 149.1 | - | 172.1 | - | 101.7 | - |  |
| Diet price / heifer (€) in year 3 | DRH3 | 88.19 | - | 174.5 | - | 169.5 | - | 193.7 | - | 155.8 | - | Assume first calving at 34, 36, 36, 36 and 35 months for systems from left to right, respectively |
| ***Vet parameters*** |  |  |  |  |  |  |  |  |  |  |  |  |
| Vet cost per cow (£) | VetCow | 96 | - | 55 | - | 70 | - | 70 | - | 107 | - | [2] |
| Cost of a vet visit (£) | VetVisit | 40 | - | 40 | - | 40 | - | 40 | - | 40 | - | Prices seen in France as reported by veterinarians to the authors |
| Cost of a vet per hour (£) | VetHour | 100 | - | 100 | - | 100 | - | 100 | - | 100 | - | Prices seen in France as reported by veterinarians to the authors |
| Cost of a caesarean | CostCes | 200 | 180-250 | 200 | 180-250 | 200 | 180-250 | 200 | 180-250 | 200 | 180-250 | Assumes 2 hours of veterinarian’s time |
| Cost of treating an abortion | CostTrAb | 30 | - | 30 | - | 30 | - | 30 | - | 30 | - | Assumes the 800kg LW cow treated with Penicillin (Longmox) [6] |
| Cost of treating a cow with clinical signs | CostCS | 35 | - | 35 | - | 35 | - | 35 | - | 35 | - | Assume farmers inject Metacam once to a cow with 700 kg |
| Cost of testing for SBV | SBVtest | 6.2 | - | 6.2 | - | 6.2 | - | 6.2 | - | 6.2 | - | UK data used as proxy [7] |
| Cost of calves vaccine | CalvesVac | 15 | 15-29 | 15 | 15-29 | 15 | 15-29 | 15 | 15-29 | 15 | 15-29 | 3 doses Clostridium, 1 dose respiratory and diarrheal vaccines |
| Cost of cow vaccine not given to culled cows | CowVac | 5 | - | 5 | - | 5 | - | 5 | - | 5 | - | Assumes cost of BVD vaccine |
| Cost of fluke control per cow (one dose) | FlukeC | 7 | - | 7 | - | 7 | - | 7 | - | 7 | - |  |
| Cost of deworming a calf | WorC | 1.3 | - | 1.3 | - | 1.3 | - | 1.3 | - | 1.3 | - | Vecoxxan and Seponver |
| **Other parameters** |  |  |  |  |  |  |  |  |  |  |  |  |
| bedding used (tonnes/cow) | BedCow | 1.5 | - | 1.15 | - | 1.25 | - | 0.5 | - | 1.8 | - | Based on 7 kg per day indoor. |
| Price of bedding (€/tonnes) | PrBed | 60 | - | 60 | - | 60 | - | 70 | - | 60 | - | [2] |
| Miscellaneous cost/cow (£) | MisCow | 65 | - | 65 | - | 96 | - | 40 | - | 85 | - | [2] |
| Discount rate | DR | 0.035 | - | 0.035 | - | 0.035 | - | 0.035 | - | 0.035 | - | The green book |

Table S4: Parameters used to simulate different beef production systems in the United Kingdom

| Parameters | Notation | Lowland autumn calving herd | | Lowland spring calving herd | | Less favoured areas autumn calving herd | | Less favoured areas spring calving herd | | References |
| --- | --- | --- | --- | --- | --- | --- | --- | --- | --- | --- |
|  |  | Value | Variability | Value | Variability | Value | Variability | Value | Variability |  |
| ***Physical performance data*** | | | |  |  |  |  |  |  |  |
| Proportion of barren cows (%) | PrpBC | 6.5 | 6.3-6.5 | 6.5 | 6.3-6.5 | 6.4 | 6.4-6.6 | 6.4 | 6.4-6.6 | [3] |
| Calves born alive per 100 cows mated | BA | 88.8 | 88.6-88.8 | 88.8 | 88.6-88.8 | 94.8 | 94.8-95.9 | 94.8 | 94.8-95.9 | [3] |
| Calves dead per 100 cows mated | CD | 2.8 | 1.7-2.8 | 2.8 | 1.7-2.8 | 2.4 | 1.6-2.4 | 2.4 | 1.6-2.4 | [3] |
| Additional mortality rate (%) of heifers used for replacement (proportion) | MH | 6 | - | 6 | - | 6 | - | 6 | - | [3] |
| Male-female calf ratio | MFR | 0.5 | - | 0.5 | - | 0.5 | - | 0.5 | - | Assumption |
| Average life of a cow (years) | LiveC | 7.5 | 7-7.5 | 8 | 7-8 | 6.5 | 6.5-7 | 7 | 7-8 | [4,8,9] |
| Average life of a bull (years) | LiveB | 5 | - | 5 | - | 4 | - | 4 | - | [4] |
| Cow mortality (%) | MrtC | 1 | - | 1 | - | 1 | - | 1 | - | [9] |
| Cow - bull ratio | CBR | 35 | - | 35 | - | 35 | - | 35 | - | [4,9] |
| Live weight of a steer at selling point (kg) | LwSteer | 375 | - | 285 | - | 360 | - | 375 | - | [8] |
| Live weight of a heifer at selling point (kg) | LwH | 340 | - | 265 | - | 330 | - | 255 | - | [8] |
| ***Animal economic data*** |  |  |  |  |  |  |  |  |  |  |
| Price per live weight of a calf (€) | PrCal | 2.33 | 2.33-2.36 | 2.33 | 2.33-2.36 | 2.33 | 2.33-2.36 | 2.33 | 2.33-2.36 | [4] |
| Cow cull value (€) | CCull | 890 | - | 890 | - | 800 | - | 800 | - | [4] |
| Price of a heifer for replacement (€) | PrH | 1,130 | - | 1,130 | - | 1,090 | - | 1,090 | - | [8] |
| Price of a bull for replacement (€) | PrB | 4,900 | - | 4,900 | - | 4,900 | - | 4,900 | - | [4] |
| Cull value of a bull | BCull | 650 | - | 650 | - | 650 | - | 650 | - | [4] |
| ***Feed parameters*** |  |  |  |  |  |  |  |  |  |  |
| Concentrate per cow (kg) | ConCow | 209 | 209-1350 | 135 | 135-670 | 209 | 209-1350 | 135 | 135-670 | [8,9] |
| Concentrate per calf (kg) | ConCal | 150 | 150-500 | 75 | 75-670 | 147 | 147-615 | 92 | 92-430 | [8,9] |
| Price of cow concentrate (€/kg) | PrConCow | 0.240 | - | 0.240 | - | 0.240 | - | 0.240 | - | [8] |
| Price of calf concentrate (€/kg) | PrConCalf | 0.258 | - | 0.258 | - | 0.258 | - | 0.258 | - | [8] |
| Cost of bulk feed/cow (€) | CostBulk | 24 | - | 22 | - | 10 | - | 20 | - | [8] |
| Forage cost (€) / hectare | CostForHa | 295 | - | 295 | - | 216 | - | 216 | - | [4] |
| Number of cows per hectare of forage | CowperHa | 1.65 | 1.4-2 | 1.8 | 1.55-2.2 | 1.25 | 1.-1.5 | 1.6 | 1.15-1.92 | [4,8] |
| ***Veterinary parameters*** |  |  |  |  |  |  |  |  |  |  |
| Vet cost per cow (€) | VetCow | 35 | 33-35 | 33 | 30-33 | 35 | 33-35 | 33 | 30-33 | [4,8] |
| Cost of a vet visit (€) | VetVisit | 37 | - | 37 | - | 37 | - | 37 | - | 10 |
| Cost of a vet per hour (€) | VetHour | 85 | - | 85 | - | 85 | - | 85 | - | [10] |
| Cost of disposing a dead animal (€) | CostDisp | 115 | 96-147 | 115 | 96-147 | 115 | 96-147 | 115 | 96-147 | Fallen stock scheme |
| Cost of a caesarean (€) | CostCes | 323 | - | 323 | - | 323 | - | 323 | - | Assuming 2 hours of veterinarian work + Anti-inflammatory injection (Rymadil (£10.83/cow)) + Anestesia (Xylazine (£1.82/cow) + Antibiotics (Alamycin LA (£1.53/cow)+ (2 meters Catgut = £4.4) [10-14] |
| Cost of treating an abortion (€) | CostTrAb | 127 | - | 127 | - | 127 | - | 127 | - | Assumes the cow is treated with Penicillin (Ultrapen), cow weight is 600kg [15] , and cost of a visit by the veterinarian (Vetvisit+Vethour) |
| Cost of treating a cow with clinical signs (€) | CostCS | 13.3 | - | 13.3 | - | 13.3 | - | 13.3 | - | Assume farmers inject Rimadyl once and cow weight is 600kg [7,14] |
| Cost of testing for SBV (€) | SBVtest | 7.6 | - | 7.6 | - | 7.6 | - | 7.6 | - |  |
| Cost of calf vaccine (€) | CalvesVac | 1.25 | - | 1.25 | - | 1.25 | - | 1.25 | - | 3 doses of Clostridium vaccination [16] |
| Cost of cow vaccine (not given to culled cows) (€) | CowVac | 0.42 | - | 0.42 | - | 0.42 | - | 0.42 | - | 1 dose of Clostridium vaccine (£0.34)  [BVD and Leptospirosis will not be saved as they are administered at insemination point] [16] |
| Cost of deworming a cow (one dose) (€) | WorC | 0.01 | - | 0.01 | - | 0.01 | - | 0.01 | - | £11.99 for 1000ml. Dose is 20ml per 50 kg. Cow weight 600kg [17] |
| Cost of deworming a calf (€) | WorCal | 0.098 | - | 0.098 | - | 0.098 | - | 0.098 | - | £419.59 for 5000ml. Dose: 1 ml per one 10kg of body weight. |
| ***Other parameters*** |  |  |  |  |  |  |  |  |  |  |
| Straw used per cow (tonnes) | BedCow | 0.8 | 0.6-0.8 | 0.7 | 0.5-0.7 | 0.85 | 0.7-0.85 | 0.75 | 0.7-0.75 | [4,8] |
| Cost of straw per tonne (€) | Strawcost | 62 | - | 62 | - | 80 | - | 80 | - | [8] |
| Miscellaneous cost / cow (€) | MisCow | 22 | 26-27 | 22 | 22-23 | 22 | 26-27 | 22 | 22-23 | [4] |
| Discount rate | DR | 0.035 | - | 0.035 | - | 0.035 | - | 0.035 | - | The green book |

Table S5: Assumptions made on general management practices and reactions to Schmallenberg virus (SBV) related disorders in beef suckler holdings in France (FR) and the United Kingdom (UK) and to estimate the impact of SBV.

| ***General management practices (without SBV)*** |
| --- |
| FR: Farms are assumed to breed all their replacement heifers.  UK: Farms are assumed to purchase all their replacement heifers. |
| ***Farmers’ reaction to clinical disease*** |
| FR-UK: Adult cattle show clinical episodes with diarrhoea, milk drop and fever  FR-UK: In case of a clinical episode, the majority of cows will not receive treatment and the veterinarian will not be called to investigate |
| ***Reproductive disorders and related management practices*** |
| FR-UK: SBV reproduction problems occur in the last trimester (stillborn or malformed calves or abortion). Due to the lack of scientific evidence of fertility problems due to SBV (e.g. early abortion, empty cows), these effects were not considered in this study.  FR-UK: A proportion of cows with reproductive disorders due to SBV will be culled as they will be considered not able to breed again or carry a calf. The meat from culled cows will be sold (cull value applied).  FR: In case of culling of cows, the only changes of costs considered are these related to extra heifer not sold and breed to replace these culled.  FR-UK: In case of late abortion, the veterinarian will be called out and antibiotic treatment will be applied  FR-UK: In some cases, the malformations will lead to dystocia and the veterinarian will be called out. In few cases of dystocia farmers will agree to conduct a caesarean.  FR-UK: When there is no dystocia, the veterinarian will not be called out and there will not be any medical treatment.  FR: The cost of culling a malformed calf is negligible. The cost of disposing a dead animal is null, since this is paid through a tax at slaughtering or normal animals.  UK: The cost of culling a malformed calf is negligible, but not the disposal cost.  FR-UK: A proportion of aborted foetuses and calves stillborn and malformed will be submitted for SBV testing |

**References**

1. Raboisson D : [Mortalité des veaux en France : quelques chiffres]. In Journées Nationales Des Groupements Techniques Vétérinaires. 14-17 May 2009. Nantes.

2. Institut Elevage Bovin viande : [Résultats 2011 des exploitations bovins viande. Résultats nationaux. Collection résultats annuels. Réseaux d’élevage pour le conseil et la prospective]. 2013:1–40.

3. EBLEX: EBLEX business pointer [http://www.eblex.org.uk/returns/business-pointers-2012/]

4. Nix J : The John Nix Farm Management Pocketbook 2013. Melton Mowbray: Agro Business Consultants Ltd; 2013.

5. Estimation second half 2012. La France Agricole n° 3443 & 3455, 2012.

6. Centravet Catalogue. Tarif général juillet 2012. [www.centravet.org]

7. Department for Environment Food and Rural Affairs: Schmallenberg virus. [http://www.defra.gov.uk/ahvla-en/disease-control/non-notifiable/schmallenberg/]

8. Agro Business Consultants: The agricultural budgeting and costing book. Melton Mowbray: ABC books; 2012.

9. Scottish Agricultural College. Farm management book 2009/2010.SAC consulting; 2013

10. Pilgrims Veterinary Practice; 2013. [http://www.pilgrimsvets.org.uk/?q=farm-veterinary-practice]

11. Anonymous. Alamycin LA Injection (100 ml). 2013 [http://www.myvetmeds.co.uk/farm/sheep-farm/antibiotics-sheep/alamycin-la-injection.htm#product-directions]

12. Anonymous. Xylazine 100mg/mL, 50 mL Vial.2013 [http://www.vetdepot.com/xylazine-100mg-ml-50-ml-vial.html]

13. Anonymous. AK Catgut Cassettes -Absorbable chromic catgut material; 2013 [http://www.animussurgical.com/product/ak-catgut-cassettes/]

14. Anonymous. Rimadyl Cattle 50mg/ml 50ml. 2013 [http://www.farmacy.co.uk/products/705-rimadyl-cattle-50mgml-50ml]

15. Anonymous. Ultrapen LA 100ml [WWW Document]. 2013 [http://www.farmacy.co.uk/products/531-ultrapen-la-100ml]

16. Anonymous. Covexin 10 100ml. 2013 [http://www.farmacy.co.uk/products/752-covexin-10-100ml]

17. Anonymous. Closamectin Pour On 2.5L [http://www.farmacy.co.uk/products/650-closamectin-pour-on-25l]
