# Supplementary material for: Application of integrated production and economic models to estimate the impact of Schmallenberg virus for various beef suckler production systems in France and the United Kingdom
Source: BMC Vet Res. 2014 Oct 26;10:254. doi: 10.1186/s12917-014-0254-z (PMC4221685; doi:10.1186/s12917-014-0254-z)
Supplement: Additional file 2: — Details results and sensitivity analysis. [file 12917_2014_254_MOESM2_ESM.doc]

Additional file 2.

Table 1: Detailed calculations and results for a beef Charolais_Calving herd in France without and with Schmallenberg virus (SBV) considering a high impact disease scenario

|  | **Charolais_Calving** | | **Calculations and comments** |
| --- | --- | --- | --- |
|  | **Without SBV** | **With SBV (high impact)** |  |
| **General model results** |  |  |  |
| Average size of herd | 100 | 100 | Assumption |
| Number of cows that calved | 93.9 | 91.9 | Average herd size *(1-Percentage of barren cows) - Number of cows with abortion |
| Number of calves at selling point | 86 | 82.2 | Average herd size * *Numeric Productivity -*  Number of cows with abortion - Number of calves stillborn, malformed or culled |
| Number of steer produced and sold | 43 | 41.1 | number of calves at selling * *Male/Female calf ratio* |
| Number of heifer produced | 43 | 41.1 | Number of calves at selling - number of steer produced |
|  |  |  |  |
| Number of heifers sold | 16.5 | 14.3 | Number of heifer produced - number of heifers needed for replacement |
| Number of Used for replacement (not sold) | 26.5 | 26.7 | Number of heifer produced - Number of heifer sold |
| Number of Heifers needed for replacement | 26.5 | 26.7 | *Replacement rate proportion +*  *Replacement rate of proportion ** *replacement heifers mortality* |
| Number of replacement heifers that died | 1.5 | 1.5 | *Replacement rate of proportion ** *replacement heifers mortality* |
| Number of heifer bought for replacement | 0 | 0 | Number of heifers needed for replacement - Number of heifer used for replacement ; if negative, equals zero |
| Number of calves that died non due to SBV | 7.4 | 7.2 | Number of cows that calved * Calf mortality rate (0-8 months) |
| Number of cows that died due to SBV | 1.5 | 1..55 | Herd size * Cow mortality rate + Number of cows with dystocia that die |
| Number of cows culled | 23.5 | 23.7 | Herd size * Replacement rate proportion /100 - Number of cows that died + Number of aborted cows that will be replaced |
| ***SBV model results*** |  |  |  |
| Number of calves stillborn, malformed or culled | 0 | 1.84 | Number of cows that calved * Proportion of calves stillborn, malformed or culled |
| Number of cows with calf stillborn, malformed or culled | 0 | 1.84 | Number of cows that calved * Proportion of calves stillborn, malformed or culled |
| Number of cows with dystocia due to SBV | 0 | 0.55 | Proportion of cows that gave birth to a malformed cow that have dystocia * Total number of cows with calf stillborn, malformed or culled |
| Number of cows with Dystocia due to SBV that require cesarean | 0 | 0.03 | Total number of cows with dystocia due to SBV * Proportion of cows with dystocia that need cesarean |
| Number of cows with SBV clinical episodes | 0 | 7.5 | Herd size * Proportion of cows with clinical episodes |
| Number of cows with SBV clinical episodes that received treatment | 0 | 0.75 | Number of cows with SBV clinical episodes * Proportion of cows with clinical episodes that require treatment |
| Number of cows with abortion | 0 | 2 | Herd size * Proportion of cows with abortions |
| Number of aborted cows that will be culled | 0 | 0.2 | Number of cows with abortion * Proportion of aborted cows that will be culled |
| Number of cows that have dystocia that die | 0 | 0.05 | Number of cows with dystocia due to SBV * Proportion of cows that die due to calving difficulties among those that have dystocia |

Table 2: Calculations of the gross margin in a beef Charolais_Calving herd in France without and with Schmallenberg virus (SBV) considering a high impact disease scenario. All values are in €.

|  | **Charolais_Calving** | | **Calculations and comments** |
| --- | --- | --- | --- |
|  | **Without SBV** | **With SBV (high impact)** |  |
| **OUTPUT** |  |  |  |
| Sales from steers | 40,557 | 37,747 | *Number of steer sold * Live weight of a steer (lw kg)* Price of steers (€/ lw kg)* |
| Sales from heifers | 14,058 | 12,192 | *Number of heifers sold * Live weight of heifers (lw kg)* Price of heifers (€/ lw kg)* |
| Sales from cows culled | 27,025 | 27,255 | *Number of culled cows * Price of a culled cow* |
| ***Sum of outputs*** | ***81,640*** | ***78,194*** |  |
| **VARIABLES COSTS** |  |  |  |
| REPLACEMENT COSTS |  |  |  |
| Cost of replacing with own heifers (feed, after weaning) | 7,348 | 7,423 | *Number of replacement heifers * Price of diet (years 1 to 3)* |
| Bull replacement cost | 500 | 500 | *Herd size * ((* *Price per bull-* *Cull value of a bull )/* *Life of a bull /* *cow:bull ratio)* |
| FEEDING COSTS |  |  |  |
| Cost of concentrates use in cows | 2,465 | 2,412 | Number of cows that calved * Concentrate per cow * Price of concentrate |
| Concentrate saved on cows that die | 0 | -1.44 | Number of cows that died due to SBV * Concentrate per cow * Price of concentrate |
| Cost of concentrate/forage used in sold steers and heifers | 4,053 | 3,780 | Concentrate per steer sold * Price of the calf concentrate * Number of steer sold + Concentrate per heifer sold * Price of the calf concentrate * Number of heifer sold |
| Cost of concentrate/forage used in replacement heifers | 1,675 | 1,692 | Concentrate per heifer sold * Price of the calf concentrate * Number of replacement heifer |
| Cost of forage per cow per year | 12,089 | 12,089 | Number of cows that calved * Forage cost/cow/year |
| VETERINARY AND MEDICINE COSTS |  |  |  |
| Vet & Med cost in cows | 9,600 | 9,600 | Herd size * Vet costs per cow |
| Vet cost saved due to vaccine/deworming in cows | 0 | -2.4 | Number of aborted cows culled * (Cost of vaccine and deworming for a cow) |
| Vet cost saved due to vaccine/deworming in calves | 0 | -63 | Number of steers and heifers not produced * (Cost of vaccine and deworming for a calf) |
| Cost veterinary assistance in cows with dystocia | 0 | 72 | Number of cows with dystocia due to SBV * Cost of a vet dirty hour |
| Cost of caesareans | 0 | 6.6 | Number of cows with caesarean * Cost of a caesarean |
| Cost of treating SBV clinical signs | 0 | 26 | Number of cows with SBV clinical episodes that received treatment * Cost of treating a SBV clinical episode |
| Cost of treating SBV abortions | 0 | 60 | Number of cows with abortion * Cost of treating an abortion |
| Cost of testing aborted foetuses and claves S/M/C for SBV | 0 | 0.6 | Number of calves stillborn, malformed or culled * Proportion of aborted foetus, that need SBV testing * Cost of testing for SBV (€) |
| OTHER COSTS |  |  |  |
| Bedding cost | 8,453 | 8,290 | Number of cows that calved * Bedding used / cow* Price of bedding |
| Miscellaneous cost | 6,105 | 5,988 | Number of cows that calved * Miscellaneous cost /cow |
| ***Sum of variables costs*** | ***52,289*** | ***51,876*** |  |
| Gross margin per herd | 29,351 | 26,318 |  |
| Gross margin (€)/cow | 293 | 263 |  |

Table 3: Economic cost due to SBV for varying input parameters for a French (FR) and British (UK) average beef suckler farm expressed as net cost (€) per cow. Values highlighted in red refer to the most likely values used in the low and high impact scenarios

|  |  | | **Percentage of stillborn and malformed calves due to SBV** | | | | | |
| --- | --- | --- | --- | --- | --- | --- | --- | --- |
|  |  |  | **0%** | **1%** | **2%** | **3%** | **4%** | **5%** |
| **FR: Charolais_Calving,** € per cow | **Percentage of cows with late abortions due to SBV** | **0%** | 0.0 | 8.4 | 16.6 | 24.8 | 32.9 | 41.1 |
|  |  | **0.5%** | 3.4 | 11.6 | 19.7 | 27.8 | 36.0 | 44.1 |
|  |  | **1%** | 6.6 | 14.7 | 22.8 | 30.9 | 39.0 | 47.0 |
|  |  | **1.5%** | 9.8 | 17.8 | 25.9 | 33.9 | 42.0 | 50.0 |
|  |  | **2%** | 13.0 | 21.0 | 29.0 | 37.0 | 45.0 | 53.0 |
|  |  | **2.5%** | 16.2 | 24.1 | 32.1 | 40.0 | 48.0 | 55.9 |
|  |  | **3%** | 19.3 | 27.3 | 35.2 | 43.1 | 51.0 | 58.9 |
|  |  | **3.5%** | 22.5 | 30.4 | 38.2 | 46.1 | 54.0 | 61.8 |
| **FR: Salers_Calving,**  € per cow | **Percentage of cows with late abortions due to SBV** | **0%** | 0.0 | 7.4 | 14.5 | 21.6 | 28.7 | 35.8 |
|  |  | **0.5%** | 3.1 | 10.2 | 17.3 | 24.3 | 31.4 | 38.5 |
|  |  | **1%** | 6.0 | 13.0 | 20.1 | 27.1 | 34.1 | 41.2 |
|  |  | **1.5%** | 8.8 | 15.8 | 22.8 | 29.8 | 36.8 | 43.8 |
|  |  | **2%** | 11.7 | 18.7 | 25.6 | 32.6 | 39.6 | 46.5 |
|  |  | **2.5%** | 14.6 | 21.5 | 28.4 | 35.3 | 42.3 | 49.2 |
|  |  | **3%** | 17.4 | 24.3 | 31.2 | 38.1 | 45.0 | 51.8 |
|  |  | **3.5%** | 20.2 | 27.1 | 34.0 | 40.8 | 47.7 | 54.5 |
| **FR: Charolais_Fattening,** € per cow | **Percentage of cows with late abortions due to SBV** | **0%** | 0.0 | 11.8 | 23.5 | 35.1 | 46.7 | 58.3 |
|  |  | **0.5%** | 5.1 | 16.7 | 28.2 | 39.8 | 51.3 | 63.0 |
|  |  | **1%** | 10.0 | 21.5 | 33.0 | 44.5 | 56.0 | 67.5 |
|  |  | **1.5%** | 14.9 | 26.3 | 37.8 | 49.2 | 60.7 | 72.1 |
|  |  | **2%** | 19.8 | 31.2 | 42.6 | 53.9 | 65.3 | 76.7 |
|  |  | **2.5%** | 24.7 | 36.0 | 47.3 | 58.7 | 70.0 | 81.3 |
|  |  | **3%** | 29.1 | 40.9 | 52.1 | 63.4 | 74.6 | 85.9 |
|  |  | **3.5%** | 34.5 | 45.7 | 56.9 | 68.1 | 79.2 | 90.5 |
| **UK : Lowland_Autumn,** € per cow | **Percentage of cows with late abortions due to SBV** | **0%** | 0.0 | 9.1 | 11.8 | 26.9 | 29.2 | 44.8 |
|  |  | **0.50%** | 4.4 | 13.3 | 22.3 | 31.1 | 40.0 | 49.0 |
|  |  | **1%** | 8.7 | 17.6 | 26.5 | 35.3 | 44.2 | 53.0 |
|  |  | **1.50%** | 13.0 | 21.9 | 30.6 | 39.5 | 48.2 | 57.1 |
|  |  | **2%** | 17.7 | 26.1 | 34.8 | 43.7 | 52.4 | 61.1 |
|  |  | **2.50%** | 21.7 | 30.4 | 39.1 | 47.9 | 56.5 | 65.2 |
|  |  | **3%** | 26.0 | 34.7 | 43.3 | 51.9 | 60.7 | 69.3 |
|  |  | **3.50%** | 30.3 | 38.9 | 47.5 | 56.1 | 64.7 | 73.3 |
| **UK : Lowland_Spring,** € per cow | **Percentage of cows with late abortions due to SBV** | **0%** | 0.0 | 7.6 | 15.1 | 22.5 | 28.9 | 37.5 |
|  |  | **0.50%** | 3.7 | 11.2 | 18.6 | 26.1 | 33.6 | 49.0 |
|  |  | **1%** | 7.4 | 11.8 | 22.1 | 29.7 | 37.0 | 44.4 |
|  |  | **1.50%** | 10.9 | 18.3 | 25.7 | 33.1 | 40.5 | 47.9 |
|  |  | **2%** | 14.6 | 22.0 | 29.3 | 36.7 | 43.9 | 51.3 |
|  |  | **2.50%** | 18.2 | 25.6 | 32.8 | 47.9 | 49.4 | 54.7 |
|  |  | **3%** | 21.4 | 29.2 | 36.4 | 43.7 | 50.9 | 58.2 |
|  |  | **3.50%** | 25.6 | 32.7 | 40.0 | 47.1 | 54.4 | 61.6 |
